# Regulation of Glucose, Fatty Acid and Amino Acid Metabolism by Ubiquitination and SUMOylation for Cancer Progression

**DOI:** 10.3389/fcell.2022.849625

**Published:** 2022-03-21

**Authors:** Shunqin Zhu, Hongyu Gu, Cheng Peng, Fanwei Xia, Huan Cao, Hongjuan Cui

**Affiliations:** ^1^ State Key Laboratory of Silkworm Genome Biology, School of Life Sciences, Southwest University, Chongqing, China; ^2^ Cancer Center, Reproductive Medicine Center, Medical Research Institute, Southwest University, Chongqing, China

**Keywords:** ubiquitination, SUMOylation, cancer, metabolic reprogramming, glucose metabolism, lipid metabolism

## Abstract

Ubiquitination and SUMOylation, which are posttranslational modifications, play prominent roles in regulating both protein expression and function in cells, as well as various cellular signal transduction pathways. Metabolic reprogramming often occurs in various diseases, especially cancer, which has become a new entry point for understanding cancer mechanisms and developing treatment methods. Ubiquitination or SUMOylation of protein substrates determines the fate of modified proteins. Through accurate and timely degradation and stabilization of the substrate, ubiquitination and SUMOylation widely control various crucial pathways and different proteins involved in cancer metabolic reprogramming. An understanding of the regulatory mechanisms of ubiquitination and SUMOylation of cell proteins may help us elucidate the molecular mechanism underlying cancer development and provide an important theory for new treatments. In this review, we summarize the processes of ubiquitination and SUMOylation and discuss how ubiquitination and SUMOylation affect cancer metabolism by regulating the key enzymes in the metabolic pathway, including glucose, lipid and amino acid metabolism, to finally reshape cancer metabolism.

## Introduction

Metabolic reprogramming occurs in many diseases, including diabetes, obesity, inflammation, cancer, and neurodegenerative diseases. Compared with normal cells, tumor cells grow malignantly and require a continuous supply of energy and nutrients, namely, biological macromolecules. The classic Warburg effect in tumor cells is defined as tumor cells undergoing glycolytic metabolism to rapidly meet energy requirements, even under aerobic conditions (Hanahan and Weinberg, 2011). This condition is considered the basic feature of tumor metabolism, which is universal in different tumor cells and is also used to diagnose and treat cancer (Moreau et al., 2017). In the pathogenesis of cancer and diabetes, the dysregulation of amino acid transporters (AATs) changes intracellular amino acid levels, leading to metabolic reprogramming and finally the occurrence of diseases (Kandasamy et al., 2018). The intricate relationship between metabolism and disease prompts us to understand and discuss the underlying mechanisms.

Ubiquitination and SUMOylation are important posttranslational modifications that control cell metabolism, signaling and differentiation. However, any abnormality in their functions leads to disease occurrence and progression (Popovic et al., 2014; Zhao et al., 2020). Genetic and epigenetic aberrations are the main cause of the dysregulation of ubiquitination and SUMOylation (Flotho and Melchior, 2013; Popovic et al., 2014). Recent studies have shown how ubiquitination and SUMOylation affect metabolic diseases. Cell metabolism is a complex and efficient process regulated by numerous proteins. Ubiquitination and SUMOylation perfectly control cell metabolism through the precise and timely regulation of substrate proteins. Here, we focus on metabolic disorders in cancer and how ubiquitination and SUMOylation affect metabolic reprogramming. Finally, we predict potential research directions and clinical applications in the future.

### The Processes of Ubiquitination and SUMOylation

**FIGURE 1:**
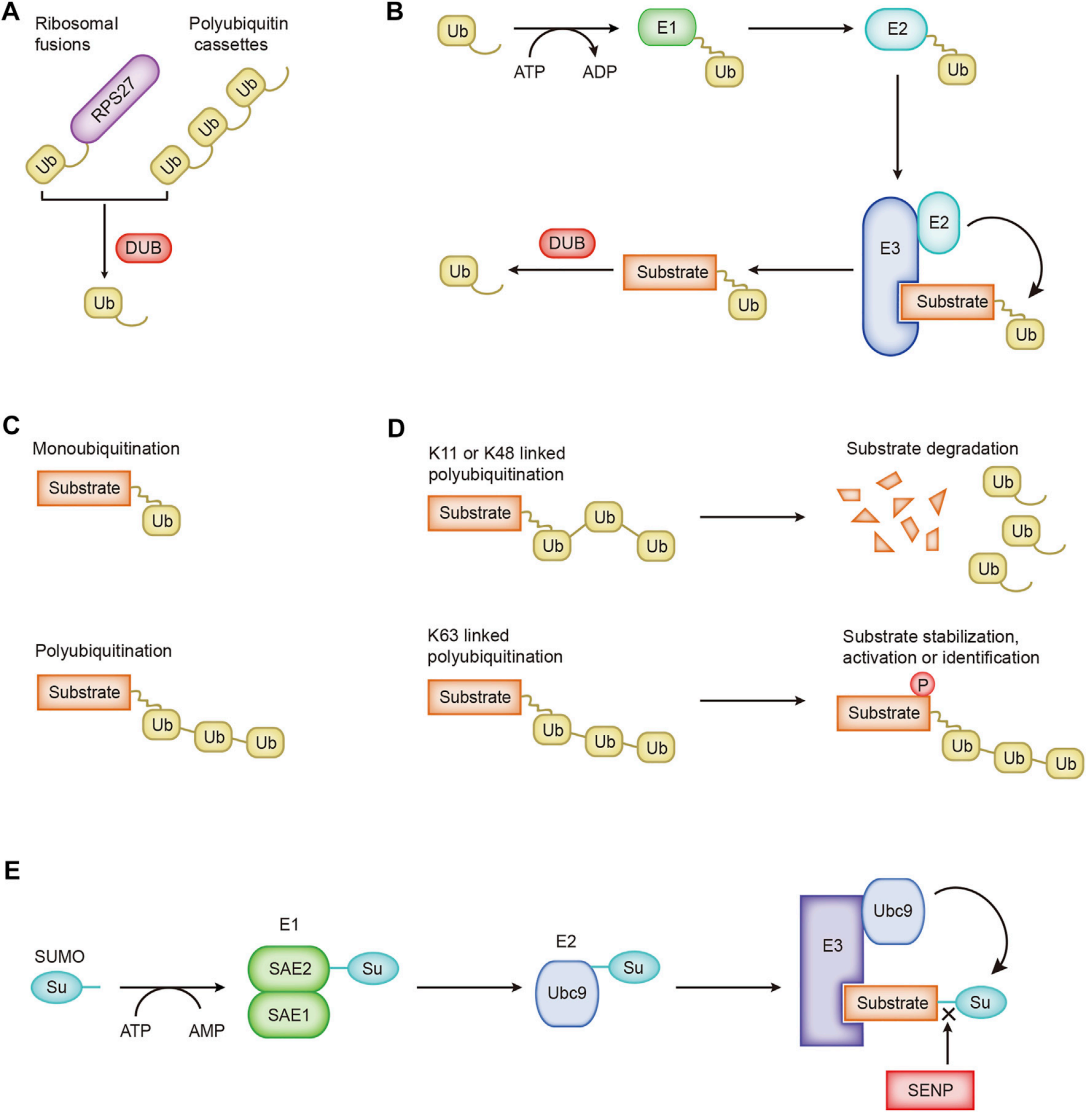
The processes of ubiquitination and SUMOylation. **(A)** Cells obtain ubiquitin from ribosomal fusions and polyubiquitin cassettes. **(B)** The cascade of ubiquitin transfer. **(C)** Different forms of ubiquitin are linked to the substrate. **(D)** Regulation of K11-, K48-, and K63-linked ubiquitination. **(E)** The SUMO cascade.

The precise communication of signals between and within cells supports the ability of cells to perform different functions and move toward different fates. Ubiquitination is the basic mechanism for establishing these signal exchanges. Ubiquitin is widely present in eukaryotic cells. It is a highly conserved protein consisting of 76 amino acids. Cells obtain ubiquitin from two sources, ribosomal fusions and polyubiquitin cassettes, which are cleaved by deubiquitinase to obtain monomeric ubiquitin molecules (Figure 1A). UBA52 and RPS27A encode ribosomal fusions, and polyubiquitin cassettes are encoded by UBB and UBC (Rape, 2018). Three enzymes participate in the ubiquitin cascade: the E1 ubiquitin-activating enzyme, E2 ubiquitin-conjugating enzyme, and E3 ubiquitin-protein ligase (Yau and Rape, 2016). The ubiquitination of the substrate usually includes three continuous processes. 1) ATP provides energy, and the ubiquitin-activating enzyme E1 activates ubiquitin to provide it a high-energy thioester. 2) Ubiquitin-activating enzyme E1 transfers the activated ubiquitin to ubiquitin-conjugating enzyme E2. 3) Ubiquitin ligase E3 transfers the ubiquitin from E2 to the substrate protein (Figure 1B). Special cases where the ubiquitin-conjugating enzyme E2 directly transfers ubiquitin to the substrate protein have also been identified. For example, the UBE2E family of E2 enzymes directly ubiquitinate the substrate SETDB1 in a manner independent of ubiquitin ligase E3, and conjugated ubiquitin is protected from active deubiquitination (Sun and Fang, 2016). Ubiquitin molecules may be cleaved from ubiquitinated proteins by deubiquitinating enzymes (DUBs) (Figure 1B). As the opposite process of ubiquitination, the process of deubiquitination plays the opposite role. Approximately 40 E2 ubiquitin-conjugating enzymes, ∼600 E3 ubiquitin ligases, and ∼100 DUBs have been identified and constitute the extremely complex and sophisticated ubiquitination/deubiquitination machinery in cells.

Ubiquitin molecules are conjugated to substrate proteins and perform different functions. Ubiquitination is divided into monoubiquitination and polyubiquitination according to the number of ubiquitin molecules attached to one lysine residue on the substrate (Figure 1C). Monoubiquitination is the simplest process. A single ubiquitin molecule is attached to one lysine of a substrate protein, which usually affects the interaction between proteins (Hicke and Dunn, 2003). Ubiquitin molecules are conjugated one after the other to form a ubiquitin chain, which eventually binds to one lysine of the substrate protein, which is polyubiquitination. Ubiquitin molecules contain seven lysines, and ubiquitin molecules are covalently connected to form different types of ubiquitin chains. Different types of ubiquitin chains lead to different fates for substrate proteins. Ubiquitin may be attached to the substrate through seven lysine residues (K6, K11, K27, K29, K33, K48, and K63) or the first methionine (M1) (Komander and Rape, 2012). K11-, K48-, and K63-linked polyubiquitination have been extensively studied. K11-linked polyubiquitination is related to the degradation of proteins during mitosis (MatSUMOto et al., 2010). K48-linked polyubiquitination-labeled proteins undergo 26S proteasome-mediated recognition and degradation, a crucial regulatory process in cells that promotes the progression of diseases such as cancer (Chen et al., 2018; Zhang et al., 2019). The ubiquitin–proteasome system participates in the degradation of more than 80% of the proteins in the cells (Glickman and Ciechanover, 2002). K63-linked polyubiquitination mediates protein stabilization, activation, and identification, which are essential for intracellular signal transduction (Figure 1D; Song et al., 2016).

A new ubiquitin-like protein called Sentrin was first reported in 1996 that binds to the death domain of FAS and regulates cell death signaling (Okura et al., 1996). A subsequent critical report named this ubiquitin-like protein SUMO (small ubiquitin-like modifier), and it covalently modifies the Ran GTPase activator protein RanGAP1 (Mahajan et al., 1997). Later, researchers adopted the name SUMO for this ubiquitin-like protein. Similar to the process of ubiquitination, SUMOylation is catalyzed by specific enzymes: SUMO-specific activating (E1), conjugating (E2), and ligating (E3) enzymes (Figure 1E). The first step is to cut the COOH termini of the SUMO protein to reveal the diglycine residues required for conjugation. A SUMO-activating enzyme contains two subunits, SAE1/Aos1 and SAE2/Uba2, conjugated with SUMO protein. ATP is hydrolyzed to generate energy for E1 and SUMO to form high-energy thioester bonds for conjugation. Next, SUMO is transferred to the only SUMO-conjugating enzyme UBC9. Finally, through catalysis by SUMO E3 ligase, SUMO is attached to the lysine residue of the substrate. Substrate proteins modified by SUMO may be unmodified by family SENPs (Sentrin/SUMO-specific proteases) (Figure 1E; Yeh, 2009). SUMO-1, SUMO-2, and SUMO-3 are the main SUMO proteins. SUMO-1 usually modifies the substrate as a monomer, while SUMO-2 and SUMO-3 form poly-SUMO chains. Monomeric SUMO or poly-SUMO chains interact with other proteins through the SUMO-interactive motif. SUMOylation changes the protein structure, leading to changes in the protein location, activity, and stability.

According to previous research, SUMOylation is a process independent of ubiquitination. However, an increasing number of studies have shown the inseparable relationship between SUMOylation and ubiquitination. These processes are jointly involved in protein degradation. The number of enzymes involved in SUMOylation is relatively small, but SUMOylation modifies various substrates. The SUMOylation pathway is related to many human diseases. An understanding of the ingenious mechanism of SUMOylation and its role in diseases may help us develop new therapeutic strategies for disease (Chang and Yeh, 2020).

## Ubiquitination, SUMOylation and Glucose Metabolism in Cancer

### Metabolic Reprogramming of Glycolysis

**FIGURE 2:**
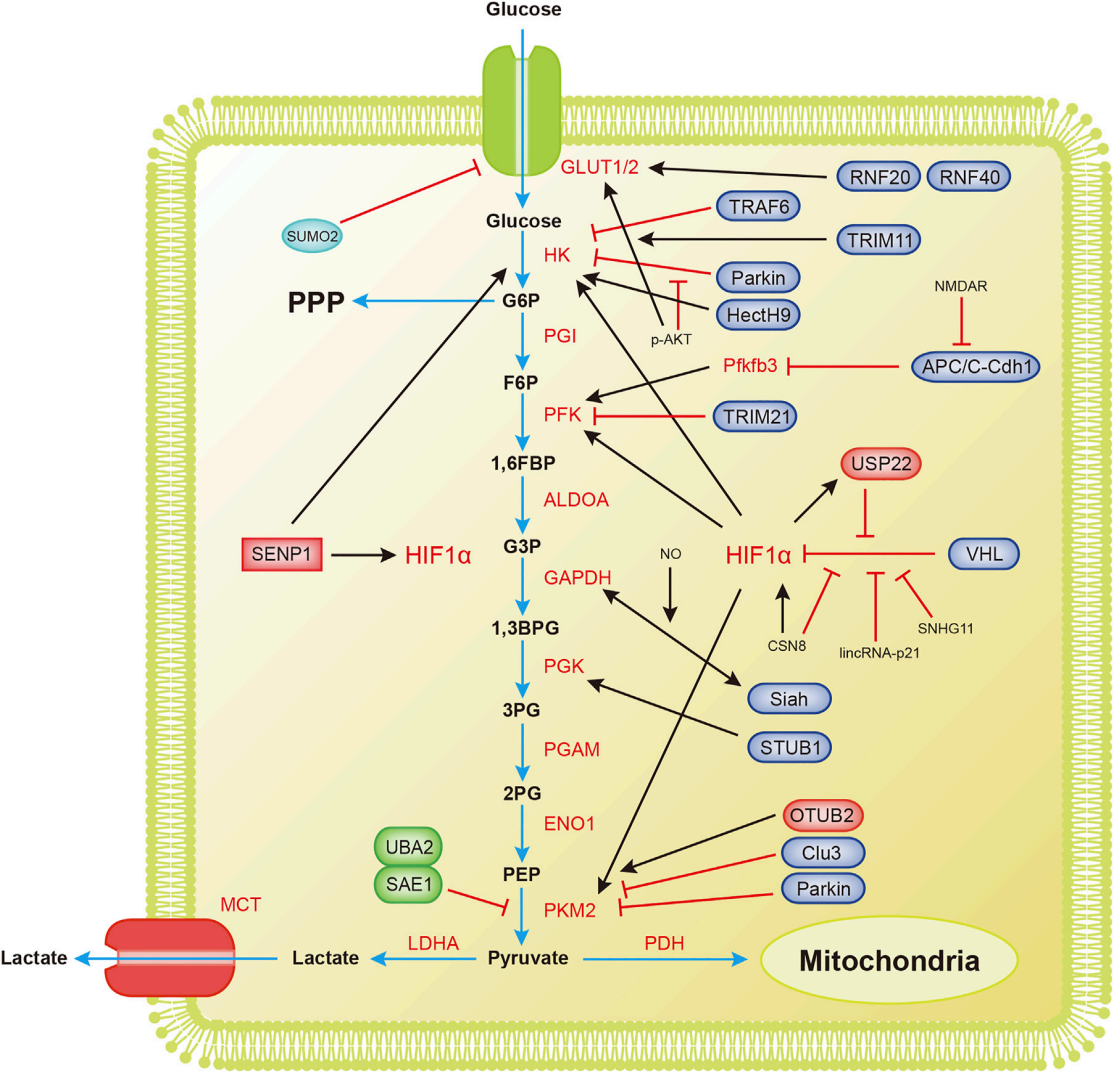
Overview of the mechanisms by which ubiquitination and SUMOylation regulate glycolysis. The blue arrow represents the flow of glycolytic substances. The black and red arrows represent activation and inhibition, respectively. 1,3BPG, 1,3-bisphosphoglycerate; 1,6FBP, fructose 1,6-biophosphate; 2PG, 3-phosphoglycerate; 3PG, 3-phosphoglycerate; ALDOA, aldose; APC/C-Cdh1, anaphase-promoting complex/cyclosome (APC/C)-Cdh1; ENO1, enolase; F6P, fructose 6-phosphate; G3P, glyceraldehyde 3-phosphate; G6P, glucose 6-phosphate; G6PDH, glucose-6-phosphate dehydrogenase; GAPDH, glyceraldehyde-3-phosphate dehydrogenase; GLUT1/2, glucose transporter 1/2; HIF1α, hypoxia-inducible factor 1α; HK, hexokinase; LDHA, lactate dehydrogenase A; MCT, monocarboxylic acid transporter; OTUB2, OTU deubiquitinating enzyme; PDH, pyruvate dehydrogenase; PFK, phosphofructokinase; Pfkfb3, 6-phosphofructo-2-kinase/fructose-2,6-bisphosphatase3; PGAM, phosphoglucomutase; PGI, glucose-6-phosphate isomerase; PGK, phosphoglycerate kinase; PKM2, pyruvate kinase M2-type; PPP, pentose phosphate pathway; RNF20/40, ring finger protein 20/40; SAE1, SUMO-activating enzyme 1; SENP1, sentrin/SUMO-specific proteases 1; STUB1, U-box containing protein 1; TPI, triode phosphate isomerase; TRAF6, TNF receptor-associated factor 6; TRIM11, tripartite motif (TRIM)-containing protein 11; TRIM21, tripartite motif (TRIM)-containing protein 21; UBA2, SUMO-activating enzyme 2; USP22, ubiquitin-specific protease 22; VHL, von Hippel-Lindau protein.

Glycolysis, one of the most important catabolic pathways in organisms, oxidizes glucose to generate pyruvate without the participation of oxygen and produces NADH and a small amount of ATP (Figure 2).

Tumor cells grow extremely fast, and their oxygen demand exceeds the oxygen supply capacity of blood vessels, resulting in a weaker ability to obtain oxygen and a subsequent state of hypoxia. The process of glycolytic reprogramming in tumor cells is described below. Hypoxia inducible transcription factor 1α (HIF-1α) is activated under hypoxic conditions, and HIF-1α enters the nucleus and interacts with hypoxia response elements to induce the expression of glycolytic enzymes and glucose transport proteins, finally accelerating glycolysis (Semenza, 2003; Weidemann and Johnson, 2008).

VHL (von Hippel-Lindau protein) is an E3 ubiquitin ligase that interacts with HIF-1α and mediates the ubiquitination of HIF-1α, leading to its degradation (Ivan et al., 2001; Jaakkola et al., 2001). The degradation of HIF-1α inhibits the metabolic reprogramming of tumors and tumor growth. Hypoxia-induced lincRNA-p21 is a hypoxia-responsive lncRNA that binds to HIF-1α and VHL, interrupting the interaction between HIF-1α and VHL and thereby reducing the degradation of HIF-1α (Wei and Lin, 2012; Yang et al., 2014). The lncRNA SNHG11 binds to the VHL recognition site on HIF-1α, thereby blocking the interaction of VHL and HIF-1α, preventing its ubiquitination and degradation, and promoting tumor metastasis (Xu et al., 2020). Under hypoxic conditions, HIF-1α transcription upregulates USP22 and TP53. USP22 promotes the stemness and glycolysis of hepatocellular carcinoma cells induced by hypoxia by deubiquitinating and stabilizing HIF-1α. Wild-type TP53, but not mutant TP53, inhibits the upregulation of USP22 induced by HIF1α. Patients with a loss-of-function mutation of TP53 have a worse prognosis (Ling et al., 2020). CSN8 increases HIF-1α mRNA expression, stabilizes the HIF-1α protein by reducing its ubiquitination, induces the epidermal-mesenchymal transition (EMT) of primary colorectal cancer cells, and increases migration and invasion (Ju et al., 2020). The SUMOylation of HIF-1α reduces its stability. SENP1 is critical for increasing the stability of HIF-1α induced by hypoxia (Cheng et al., 2007). Hypoxia enhances the stemness of hepatocellular carcinoma cells and hepatocarcinogenesis by enhancing the deSUMOylation of HIF-1α by SENP1 and increasing the stability and transcriptional activity of HIF-1α (Cui et al., 2017).

Hexokinase (HK) is the first rate-limiting enzyme in glucose metabolism. Parkin ubiquitin ligase ubiquitinates HK1 (Okatsu et al., 2012). Parkin is a critical protein in the tumor suppressor pathway and a stress-activated effector. It inhibits the proliferation and metastasis of malignant cells by interfering with metabolic reprogramming. This reprogramming includes the ubiquitination of HK1, which affects the glycolysis process (Agarwal et al., 2021). Activated AKT inhibits HK1 ubiquitination, thereby promoting tumorigenesis. The long noncoding RNA LINC00470 positively regulates the activation of AKT in glioblastoma, inhibits HK1 ubiquitination, and leads to glycolytic reprogramming (Liu et al., 2018). HK2 is an essential regulator of glycolysis that couples metabolism and proliferation activities in cancer cells. The E3 ubiquitin ligase TRAF6 modifies HK2 by K63 ubiquitination, a process that is important for the recognition of HK2 by the autophagy receptor protein SQSTM1/p62 and subsequent selective autophagic degradation. This result reveals the relationship between autophagy and glycolysis in liver cancer (Jiao et al., 2018). The nonproteolytic ubiquitination of HK2 by HectH9 regulates the mitochondrial localization and function of HK2. Loss of HectH9 inhibits HK2, thereby hindering tumor glucose metabolism and growth (Lee et al., 2019). HK2 is SUMOylated at K315 and K492 and deSUMOylated by SENP1. HK2 lacking SUMO prefers to bind to mitochondria, increasing glucose consumption and lactate production and reducing mitochondrial respiration. This metabolic reprogramming strengthens prostate cancer cell proliferation and chemotherapy resistance (Shangguan et al., 2021).

PFK1 is the second rate-limiting enzyme in glycolysis. With the participation of ATP, it catalyzes the production of fructose 1,6-bisphosphate from 6-phosphofructose. E3 ubiquitin ligase tripartite motif (TRIM)-containing protein 21 (TRIM21) targets PFK1 for ubiquitination and degradation. Cells transfer from stiff to a soft substrate, and the disassembly of stress fibers releases TRIM21, which degrades PFK1 and subsequently reduces the rate of glycolysis. However, transformed non-small cell lung cancer cells maintain high levels of glycolysis by downregulating TRIM21 and isolating residual TRIM21 in the substrate-insensitive stress-fiber subset, regardless of the changing environmental mechanics (Park et al., 2020).

Pyruvate kinase M2 (PKM2) is the catalytic enzyme required for the last step of glycolysis and the third rate-limiting enzyme. Parkin mediates the ubiquitination of PKM2 and reduces its enzymatic activity (Liu et al., 2016). Overexpression of cannabinoid receptor-interacting protein 1 (CNRIP1) activates Parkin, leading to PKM2 degradation and thereby promoting cell growth and metastasis in intrahepatic cholangiocarcinoma (ICC) (Chen D. et al., 2021). OTUB2, an OTU deubiquitinating enzyme, directly interacts with PKM2. It promotes glycolysis by preventing Parkin from ubiquitinating PKM2 and enhancing the activity of PKM2 (Yu et al., 2021). Studies have found that PKM2 regulates apoptosis and promotes tumor proliferation. PKM2 translocates to mitochondria in response to oxidative stress, and it phosphorylates Bcl2 at threonine (T) 69. Phosphorylated Bcl2 prevents its ubiquitination and degradation by Cul3-based E3 ligase (Liang et al., 2017). SUMO-activating enzyme 1 (SAE1) and SUMO-activating enzyme 2 (UBA2) mediate the SUMOylation of PKM2, which then promotes its phosphorylation and nuclear localization, reduces its pyruvate kinase (PK) activity and promotes glycolysis (Wang et al., 2020b). PKM2, which undergoes SUMOylation at lysine 270 (K270), triggers a conformational change from a tetramer to a dimer. This conformational change reduces the PK activity of PKM2 and leads to the nuclear translocation of PKM2. SUMO-modified PKM2 recruits RUNX1 and promotes its degradation through the SUMO-interacting motif, which inhibits the myeloid differentiation of NB4 and U937 leukemia cells (Xia et al., 2021).

Other enzymes in the glycolysis process are also ubiquitinated and/or SUMOylated, profoundly affecting glycolysis. 6-Phosphofructo-2-kinase/fructose-2,6-bisphosphatase 3 (Pfkfb3) generates fructose-2,6-bisphosphate, which strongly activates PFK1 and promotes glycolysis. Pfkfb3 in neurons is ubiquitinated by the E3 ubiquitin ligase anaphase-promoting complex/cyclosome (APC/C)-Cdh1, causing Pfkfb3 to be degraded by proteases (Almeida et al., 2010; Zhu et al., 2019). Astrocytes have low APC/C-Cdh1 activity and therefore express Pfkfb3 at higher levels than neurons (Herrero-Mendez et al., 2009). A report has shown that glutamate receptors (NMDARs) stabilize Pfkfb3 by inhibiting APC/C-Cdh1, thereby changing neuronal metabolism and leading to oxidative damage and neurodegeneration (Rodriguez-Rodriguez et al., 2012). Interestingly, lysine demethylase KDM2A was recently reported to target Pfkfb3 for ubiquitination and degradation as a process to inhibit the proliferation of multiple myeloma (Liu et al., 2021). GAPDH is an abundant protein involved in glycolysis and catalyzes the conversion of glyceraldehyde 3-phosphate to glycerate 1,3-diphosphate. Activated nitric oxide (NO) nitrosylates GAPDH. This nitrosylation inhibits the catalytic activity of GAPDH and allows it to bind Siah, an E3 ubiquitin ligase. The GAPDH/Siah protein complex enters the nucleus and degrades the Siah target protein to cause cell death (Hara et al., 2006). Phosphoglycerate kinase 1 (PGK1) is related to the progression of many cancers and is an important enzyme in glycolysis. The E3 ubiquitin ligase STUB1 targets PGK1 for ubiquitination and subsequent degradation. The lncRNA LINC00926 promotes STUB1-mediated ubiquitination of PGK1 to downregulate PGK1 expression, thereby suppressing the growth of breast cancer (Chu et al., 2021). Interestingly, another lncRNA, GBCDRlnc1, inhibits the ubiquitination of PGK1 and endows gallbladder cancer cells with chemotherapy resistance by activating autophagy (Cai et al., 2019).

Glucose transport is also important for glycolysis. Glucose transporters GLUT1 and GLUT2 mediate glucose transport, and glycolytic reprogramming will also alter the expression of GLUT1 and GLUT2. In response to EGF, the E3 ligase Skp2 mediates the nonproteolytic ubiquitination modification of AKT, thereby activating the AKT pathway and promoting GLUT1 expression and glycolysis (Chan et al., 2012). According to a recent study, the circular RNA circRNF13 binds to the 3′-UTR of the SUMO2 gene and prolongs the half-life of the SUMO2 mRNA. Upregulated SUMO2 promotes GLUT1 degradation through its SUMOylation and ubiquitination, which inhibits glycolysis and ultimately inhibits the proliferation and metastasis of nasopharyngeal carcinoma (Mo et al., 2021). Hypoxia-induced SUMOylation of HIF1α also promotes the expression of GLUT1 (Cheng et al., 2007). The E3 ubiquitin ligase TRIM11 promotes glycolysis in breast cancer by participating in the AKT/GLUT1 signaling pathway (Song et al., 2019). E3 ubiquitin ligases ring finger protein 20 (RNF20) and RNF40 target and regulate GLUT2 levels to affect islet β-cell function (Wade et al., 2019).

### Metabolic Reprogramming of the Tricarboxylic Acid (TCA) Cycle

**FIGURE 3:**
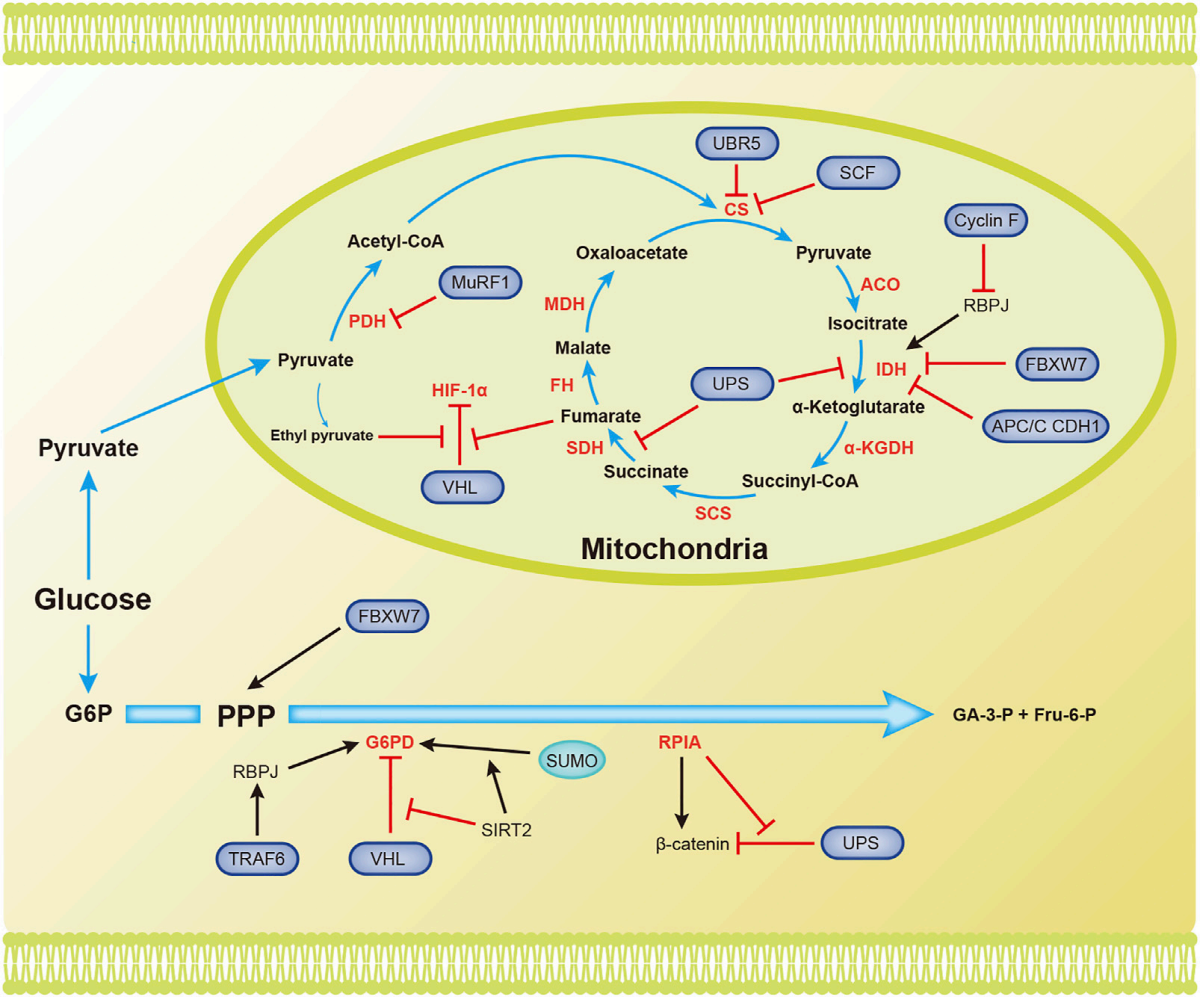
Overview of the mechanisms by which ubiquitination and SUMOylation regulate the TCA cycle and PPP. The blue arrow represents the flow of metabolites. The black and red arrows represent activation and inhibition, respectively. ACO, aconitase; CS, citrate synthase; FBW7, F-box and WD repeat domain-containing protein 7; FH, fumarate hydratase; Fru-6-P, fructose 6-phosphate; G6PD, glucose-6-phosphate dehydrogenase; GA-3-P, glyceraldehyde 3-phosphate; IDH, isocitrate dehydrogenase; MDH, malate dehydrogenase; PDH, pyruvate dehydrogenase; RPIA, ribose-5-phosphate isomerase A; SCF, Skp1-Cul1-F-box protein; SCS, succinyl-CoA synthetase; SDH, succinate dehydrogenase (Complex II); SIRT2, silent information regulator 2; SM22α, smooth muscle (SM) 22α protein; UPS, ubiquitin proteasome system; α-KGDH, α-ketoglutarate dehydrogenase.

Pyruvate is converted to acetyl-CoA, which then enters the TCA cycle. The TCA cycle requires the presence of oxygen. Acetyl-CoA is completely oxidized in the TCA cycle to generate NADH, FADH2, CO_2_, and GTP (Figure 3).

Several enzymes in the TCA cycle function as tumor suppressors and inhibit the progression of tumor cells. Their ubiquitination and/or SUMOylation affect their functions, thereby reducing their suppressive effects on tumors. The ubiquitin ligase MuRF1, muscle-specific RING-finger protein 1, interacts with PDH to regulate its stability (Hirner et al., 2008). Citrate synthase (CS) is posttranscriptionally regulated by UBR5-mediated ubiquitination (Peng et al., 2019). SCF (Ucc1) ubiquitin ligase also mediates the ubiquitination and degradation of CS by proteases (Nakatsukasa et al., 2015). Isocitrate dehydrogenase 1 and 2 (IDH1/2) catalyze the oxidative decarboxylation of isocitrate to α-ketoglutarate (α-KG), and recurrent mutations in these genes have been confirmed in glioblastoma and acute myeloid leukemia (Dang et al., 2010). Cyclin F, the substrate recognition subunit of the Skp1-Cul1-F-box protein (SCF) E3 ubiquitin ligase complex, mediates the polyubiquitination of RBPJ at Lys315 and subsequent degradation under metabolic stress. RBPJ regulates IDH expression, regardless of IDH mutation. Therefore, Cyclin F attenuates the carcinogenic ability of IDH by reducing the expression of RBPJ (Deshmukh et al., 2018). Succinate dehydrogenase (SDH) catalyzes the production of fumaric acid from succinate. Decreased SDH activity will cause succinate to accumulate in cells, and succinate inhibits HIF-α hydroxylases. This inhibition will cause the E3 ligase pVHL to dissociate from HIF-1α and ultimately maintain the stability and activation of HIF-1α (Selak et al., 2005). F-box and WD repeat domain containing 7 (FBXW7), another SCF E3 ubiquitin ligase substrate recognition element, is inversely related to the expression of IDH1 in gliomas. Deletion of FBXW7 significantly increases IDH1 expression by inhibiting the degradation of sterol regulatory element binding protein 1 (SREBP1). This process weakens the cellular buffering capacity against radiation-induced oxidative stress and enhances radiation sensitivity (Yang et al., 2021). IDH2 is an enzyme that produces NADPH, and NADPH blocks ROS in cells. APC/C CDH1 mediates the ubiquitination of IDH2 and contributes to the increase in ROS levels during mitosis (Lambhate et al., 2021). Succinate dehydrogenase subunit A (SDHA) is specifically ubiquitinated in organelles by the ubiquitin proteasome system (UPS). Inhibition of UPS-mediated SDHA ubiquitination and degradation promotes the production of ATP, malate, and citrate (Lavie et al., 2018). SDH5 depletion inhibits p53 degradation through the ubiquitin/proteasome pathway, thereby promoting apoptosis and increasing the radiosensitivity of NSCLC cells (Zong et al., 2019). Ethyl pyruvate, a derivative of pyruvate, also inhibits pVHL-mediated degradation of HIF-1α (Kim et al., 2010). Fumarase is an enzyme in the TCA cycle that participates in DNA repair in cells. The ubiquitination of lysine 79 in fumarase inhibits its function in the TCA cycle and DNA damage repair. This report reveals how posttranslational modifications affect fumarase function (Wang S. et al., 2020). Tumor cells use Gln in the TCA cycle to maintain biosynthesis and support rapid growth and proliferation. The ubiquitin ligase RNF5 interacts with the L-glutamine carrier proteins SLC1A5 and SLC38A2 (SLC1A5/38A2) to mediate their ubiquitination and degradation. This degradation reduces the uptake of Gln and components of the TCA cycle, leading to autophagy and cell death (Jeon et al., 2015). To the best of our knowledge, few studies have assessed the involvement of SUMOylation in regulating the TCA cycle.

### Metabolic Reprogramming of the Pentose Phosphate Pathway

In addition to the final production of NADPH, glyceraldehyde 3-phosphate (GA-3-P), and fructose 6-phosphate (Fru-6-P), the pentose phosphate pathway also generates many intermediates that provide raw materials for cellular biosynthesis (Figure 3).

Glucose-6-phosphate dehydrogenase (G6PD) is the rate-limiting enzyme in the PPP and is important for maintaining NADPH levels. High glucose concentrations promote the degradation of G6PD through pVHL-mediated ubiquitination of G6PD, which leads to ROS accumulation and podocyte injury (Wang M. et al., 2019). TRAF6 mediates K63 ubiquitination of the smooth muscle (SM) 22α protein, promoting the interaction between SM22α and G6PD. Ubiquitinated SM22α enhances the activity of G6PD by mediating the membrane translocation of G6PD (Dong et al., 2015). According to a recent study, silent information regulator 2 (SIRT2) interacts with G6PD to increase its activity through deacetylation, leading to a decrease in ubiquitination and an increase in SUMOylation to increase G6PD stability (Ni et al., 2021). The lack of the E3 ubiquitin ligase FBW7 reduces substrate flux through the PPP and accelerates the production of reactive oxygen species (ROS) in macrophages (Wang et al., 2020a). The dysregulation of ribose-5-phosphate isomerase A (RPIA) in the PPP promotes liver, lung, and breast tumorigenesis. Unlike its function in the PPP, RPIA enters the nucleus to form a complex with β-catenin, and this interaction prevents the ubiquitination and subsequent degradation of β-catenin in colorectal cancer. This mechanism explains the role of RPIA in promoting tumorigenesis (Chou et al., 2018).

## Ubiquitination and SUMOylation in Lipid Metabolism in Cancer

**FIGURE 4:**
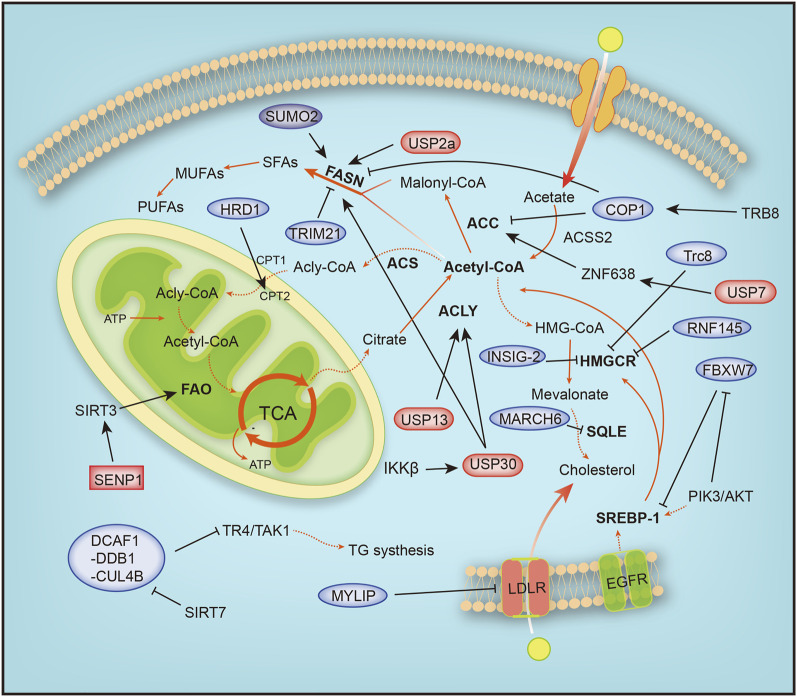
Overview of the mechanisms by which ubiquitination and SUMOylation regulate lipometabolism. ACC, acetyl-CoA carboxylase; ACLY, ATP citrate lyase; ACS, acyl-CoA synthetase; ACSS2, acyl-CoA synthetase short-chain family member 2; ATP, adenosine triphosphate; COP1, constitutive photomorphogenic protein 1; CPT1, carnitine palmitoyl-transferase1; CPT2, carnitine palmitoyl-transferase 2; DCAF1-DDB1-CUL4B, DDB1-CUL4-associated factor 1 (DCAF1)/damage-specific DNA binding protein 1 (DDB1)/cullin 4B; EGFR, epidermal growth factor receptor; FAO, fatty acid oxidation; FASN, fatty acid synthase; HMG-CoA, 3-hydroxy-3-methylglutaryl-coenzyme A; HMGCR, 3-hydroxy-3-methylglutaryl-coenzyme A reductase; HRD1, HMG-CoA reductase degradation protein 1; INSIG-2, insulin-induced gene 2; LDLR, low-density lipoprotein receptor; MARCH6, membrane-associated RING-CH 6; MUFA, monounsaturated FA; MYLIP, myosin regulatory light chain-interacting protein; PIK3, phosphoinositide-3-kinase; PUFA, polyunsaturated FA; RNF145, ring finger protein 145; SENP1, SUMO-specific protease 1; SFA, saturated FA; SIRT1, silent information regulator 1; SIRT3, silent information regulator 3; SQLE, squalene epoxidase; SREBP1/2, sterol regulatory element-binding protein 1/2; SUMO2, small ubiquitin-like modifier 2; TCA, tricarboxylic acid cycle; TR4/TAK1, testicular nuclear receptor 4; Trc8, translocation in renal carcinoma chromosome 8 gene; Trim 21, tripartite motif 21; USP13, ubiquitin specific protease 13; USP2a, ubiquitin specific protease 2a; USP30, ubiquitin specific protease 30, USP7, ubiquitin specific protease 7; ZNF638, zinc finger protein 638.

Eukaryotic cells degrade proteins through two pathways, autophagy and the ubiquitin–proteasome system (UPS), which are responsible for 10–20% and 80–90% of intracellular protein hydrolysis, respectively (Ciechanover and Kwon, 2015). The UPS is responsible for the selective hydrolysis of proteins by adding ubiquitin molecules to the substrate and degrading it in the proteasome. Ubiquitination is crucial for lipid metabolism. In recent studies, a correlation between the role of ubiquitin in the signaling pathways of lipid metabolism and the effect of the ubiquitin system on the development of human cancer has been identified. Lipometabolism includes fatty acid oxidation (FAO), fatty acid synthesis (FAS), cholesterol metabolism, ketone body metabolism and acetate metabolism. Acetyl-CoA is the substrate for *de novo* synthesis of fatty acids, and intracellular ubiquitination includes proteolytic and nonproteolytic pathways. In addition to controlling protein stability, it is crucial for regulating lipid metabolism homeostasis, inflammation, autophagy, and DNA damage repair (Popovic et al., 2014). The uncontrollable ubiquitin system abnormally activates or inhibits certain cellular metabolic pathways, thus affecting cancer development. Next, we focused on how ubiquitination and SUMOylation affect cancer by affecting the intracellular lipid metabolism (Figure 4).

### Fatty Acid Metabolic Reprogramming

The proliferation of cancer cells requires a large amount of material and energy, during which the metabolism of cells is reprogrammed. The TCA cycle is characterized by condensing acetyl coenzyme A and oxaloacetic acid to generate citric acid, anaplerosis of oxaloacetic acid, the production of intermediate products, and the energy release process (Figure 4). Glutamine is a precursor of mitochondrial oxaloacetic, supplying oxaloacetic acid required for the TCA cycle. Highly invasive ovarian cancer (OVCA) cells depend on glutamine, and glutamine is transformed into α-ketoglutarate (α-KG or 2-oxoglutarate). α-KG is reduced to isocitrate by isocitrate dehydrogenase and regenerated into citric acid. Citric acid produced from glutamine is transported into the cytoplasm to produce acetyl-CoA and participate in fatty acid synthesis (Metallo et al., 2011; Mullen et al., 2011). ACLY is expressed at the highest levels in the liver and white adipose tissue, and ACLY is upregulated and activated in a variety of tumor tissues (Chypre et al., 2012). Overexpression of USP13 is associated with malignant development and a poor prognosis of OVCA, and USP13 deubiquitinates and stabilizes ACLY. The decomposition of glutamine, ATP production, and fatty acid synthesis are accelerated. USP13 inhibition significantly blocks acetyl-CoA production and OVCA proliferation (Han et al., 2016). Strategies targeting USP13 have been proposed as a potential treatment for OVCA.

Fatty acid oxidation is a complex process characterized by a multistep reaction, and fatty acids are acylated by acyl-CoA synthase to form acyl-CoA on the endoplasmic reticulum and mitochondrial outer membrane. The oncoprotein Src is activated via autophosphorylation by two carnitine fatty acid transferases (CPT1 and CPT2) that enter the mitochondrial matrix and undergo fatty acid β oxidation. HMG-CoA reductase degradation protein 1 (HRD1), an E3 ubiquitin ligase, controls cholesterol production and regulates FAO metabolism by regulating the key rate-limiting enzyme HMG-COA reductase (HMGCR). In glutamine-deficient TNBC, HRD1 expression is downregulated, and CPT2 is stabilized by K48-linked ubiquitination, leading to increased FAO metabolism. Inhibition of CPT2 expression significantly reduces the proliferation of TNBC cells (Hampton and Rine, 1994; Hampton et al., 1996). In addition, CB839, a glutaminase (GLS) inhibitor, effectively treated TNBC cells with high HRD1 expression (Guo et al., 2021). Tumor cell metabolism is accompanied by reprogramming, as evidenced by increased glutamine and glucose uptake. Triple-negative breast cancer (TNBC) is a heterogeneous subtype of breast cancer with a high local recurrence rate and high risk of metastasis. Dysregulation of fatty acid oxidation is often associated with the progression of TNBC (Sun et al., 2020). TNBC has the metabolic characteristics of dependence on glutamine and accelerated fatty acid oxidation (FAO). When cells grow rapidly, glutamine consumption increases, resulting in a local glutamine deficiency. While fatty acids may be used as alternative energy sources for cell growth, studies have found that inhibiting MYC-dependent FAO in TNBC is an important target for the treatment of TNBC (Camarda et al., 2016).

Lipid metabolic reprogramming also frequently occurs in liver cancer, and lipid synthesis and metabolism are increased during liver cancer. The mitochondrial deubiquitination enzyme USP30 plays an important role in hepatocellular carcinoma (HCC) driven by high fat diets (HFDs). USP30 is phosphorylated and stabilized by IκB kinase β (IKKβ), which promotes USP30 deubiquitination and stabilizes ACLY and fatty acid synthase (FASN) to induce lipid generation and tumorigenesis. USP30 inhibitors significantly inhibit lipid synthesis and tumorigenesis (Gu et al., 2021). USP7 plays an important role in HCC caused by aberrant *de novo* lipogenesis (DNL). The expression of ubiquitin-specific peptidase 7 (USP7), a deubiquitinating enzyme, is positively correlated with malignant cancer by deubiquitinating MDM2, which inhibits the activation of P53 (Kon et al., 2010). USP7 deubiquitinates and stabilizes zinc finger protein 638 (ZNF638), a zinc finger protein, and promotes the transcription of ZNF638 by stabilizing cAMP-responsive element-binding protein (CREB). The USP7/ZNF638 axis activates AKT/mTORC1/S6K signaling, promotes the accumulation of cleaved-SREBP1C, and deubiquitinates nuclear cleaved-SREBP1C. Next, the USP7-ZNF638-cleaved-SREBP1C complex upregulates the expression of acetyl-CoA carboxylase (ACACA), FASN, and stearoyl-CoA desaturase (SCD) to enhance DNL and tumorigenesis (Ni et al., 2020).

Abnormal fat formation is associated with a number of malignant features, including clinically increased tumor invasion, activation of the AKT signaling pathway, and inhibition of adenosine monophosphate-activated protein kinase. The AKT-mTORC1-RPS6 signaling pathway enhances lipogenesis to promote the HCC process. In cancer, unconstrained lipogenesis is necessary to maintain a steady supply of lipids and lipid precursors that facilitate membrane production and postlipid transformation. AKT overactivation leads to increased lipid biosynthesis and higher levels of lipid-producing proteins. The AKT/mTORC1 pathway induces adipogenesis through transcriptional and posttranscriptional mechanisms. AKT promotes *de novo* adipogenesis by blocking the proteasomal degradation of SREBP1, SREBP2, and FASN. SREBP1 is an important transcription factor involved in fatty acid metabolism that upregulates fatty acid synthesis and extracellular lipid uptake. AKT inhibits SREBP1 and SREBP2 ubiquitination by inhibiting the E3 ligase FBXW7 after phosphorylation by GSK-3β to activate the expression of lipid metabolism genes (Sundqvist et al., 2005; Tu et al., 2012; Zhao et al., 2012). In addition, SIRT1-mediated deubiquitination of SREBP promotes its ubiquitination and degradation (Walker et al., 2010). Moreover, AKT also upregulates USP2a, a deubiquitination enzyme, and blocks the degradation of FASN. In summary, ubiquitination and SUMOylation are important effector mechanisms of the AKT/mTORC1 axis in human HCC (Calvisi et al., 2011). PTEN is a tumor suppressor that inhibits the PI3K/AKT signaling pathway and is modified by ubiquitylation, SUMOylation and phosphorylation in the process of PTEN function and nuclear transport. PTEN neddylation is promoted by XIAP ligase and removed by NEDP1 deneddylase. Lys197 and Lys402 are the main neddylation sites of PTEN. In contrast, neddylated PTEN promotes tumor proliferation and metabolism by dephosphorylating the FASN protein, inhibiting trim21-mediated ubiquitylation and degradation of FASN, and promoting *de novo* fatty acid synthesis. This phenomenon suggests that neddylation transforms PTEN from a cancer suppressor to an oncogene (Lee et al., 2018; Xie et al., 2021).

During fatty acid synthesis, acetyl-CoA is utilized only after activated acetyl-CoA carboxylase (ACC), a rate-limiting enzyme in fatty acid synthesis, is degraded by phosphorylation and ubiquitination by increasing catecholamine levels during fasting. The phosphorylated form is inhibited, and the dephosphorylated form is activated. Tribbles 3 (TRB3) induces ACC degradation to inhibit fatty acid synthesis and promote oxidative lipid decomposition, and TRB3 interacts with E3 ubiquitin ligase constitutive photomorphogenic protein 1 (COP1) to promote ACC degradation (Qi et al., 2006).

FASN is a key enzyme involved in the synthesis of fatty acids. FASN is expressed at relatively low levels in normal tissues, but it is abnormally upregulated in tumors. The synthesis of fatty acids affects the metabolism of sugar, fat and cholesterol by activating PPARα (Chakravarthy et al., 2005). SHP2, a tyrosine phosphatase with an SH2 domain, interacts with the E3 ubiquitin ligase COP1 to regulate the ubiquitination of FASN. Moreover, SHP2 also regulates the expression of SREBP1c, which directly activates FASN gene expression via the PI3K-AKT signaling pathway (Yu et al., 2013). In addition, overactivation of the PI3K-Akt signaling pathway is accompanied by overactivation of ErbB1 (epidermal growth factor receptor) or ErbB2 (HER2/NEU) and abnormal expression of FASN. FASN inhibitors significantly increase the sensitivity of cells to anti-ErbB drugs by promoting the ubiquitination and degradation of PI3K effector proteins (Tomek et al., 2011). On the one hand, these treatments inhibit lipid synthesis in tumor cells, and on the other hand, they block PI3K signaling in ovarian cancer. Therefore, tumor therapy targeting FASN is a promising. SIRT7 binds to the DDB1-CUL4-associated factor 1 (DCAF1)/damage-specific DNA binding protein 1 (DDB1)/cullin 4B (CUL4B) E3 ubiquitin ligase complex to block the degradation of TR4/TAK1, resulting in increased synthesis and storage of fatty acids (Yoshizawa et al., 2014; Zhu et al., 2019). In breast cancer cell lines, SUMOylation prevents FASN degradation by the proteasome, and the reduction in SUMOylation caused by SUMO2 silencing reduces the stability of FASN and inhibits the development of tumor cells (Floris et al., 2020).

In addition, ubiquitination plays a crucial role in other diseases of the human immune system. Fatty acid metabolism includes cytoplasmic fatty acid synthesis (FAS) and mitochondrial fatty acid oxidation (FAO). Inducible regulatory T (iTreg) cells inhibit excessive or abnormal immune responses and are essential for maintaining immune homeostasis. Functional defects in Tregs may lead to autoimmune diseases, such as inflammatory bowel disease (IBD) and systemic lupus erythematosus (SLE). Differentiation of activated T (Th0) cells into inducible regulatory T (iTreg) cells requires cellular metabolic reprogramming from fatty acid synthesis to fatty acid oxidation. In the differentiation of inducible regulatory T cells stimulated by TGF-β signaling, ATP-citrate lyase (ACLY) is ubiquitinated by Cul3-KLHL25. ACLY degradation promotes iTreg cell differentiation by promoting cellular fatty acid oxidation and maintaining the stability of the immune system to prevent the occurrence of autoimmune diseases. In addition, ACLY transforms citrate into acetyl-CoA and oxaloacetate, providing acetyl-CoA substrates for *de novo* fatty acid synthesis. Ubiquitination of ACLY inhibits *de novo* fatty acid synthesis, resulting in impaired cell proliferation (Tian et al., 2021). Sirt3 regulates the mitochondrial adaptation to metabolic stress, and SUMOylation inhibits Sirt3 in mitochondria. When cells starve, Sirt3 is deSUMOylated by SENP1, a SUMO-specific protease that activates Sirt3 and increases fatty acid metabolism (Wang T. et al., 2019).

### Cholesterol Metabolic Reprogramming

Cholesterol plays a vital role in maintaining cell homeostasis. It is most abundant in the eukaryotic plasma membrane, regulates cell membrane fluidity and material transport and is a precursor of intermediate metabolites such as bile acids and steroids (Haines, 2001; Hannich et al., 2011). Cholesterol metabolism is strictly regulated in the human body. A single disorder blocks arteries and causes heart attacks and strokes. Our body controls the amount of cholesterol through an enzyme called 3-hydroxy-3-methylglutaryl-coenzyme A reductase (HMGCR). In the next sections, we will focus on the functions of ubiquitination and SUMOylation in cholesterol metabolism (Figure 4).

HMGCR is the rate-limiting enzyme in cholesterol and nonsteroidal isoprene biosynthesis and the therapeutic target of statins (Sun et al., 2020). When cholesterol levels in cells are reduced, sterol response element binding proteins (SREBPs) bind to SREs in the promoter region of HMGCR to promote its transcription (Osborne, 1991), and the HMGCR mRNA and protein half-lives are extended, ensuring the supply of mevalonate (Goldstein and Brown, 1990). RNF145, a sterol-responsive-resident E3 ligase, gradually accumulates with depletion of sterols and sensitively responds to the cholesterol level. Reduced cellular cholesterol levels, the formation of RNF145, increased cellular cholesterol levels, and RNF145 mediate HMGCR ubiquitination and proteasomal degradation. Alternatively, in the presence of an RNF145 and GP78 deficiency, uBE2G2 ligase 1 partially regulates HMGCR activity (Loregger et al., 2017; Jiang et al., 2018; Menzies et al., 2018). Hypoxia-induced expression of insulin-induced gene 2, insig-2, inhibits cholesterol synthesis by mediating sterol-induced ubiquitination and HMGCR degradation (Hwang et al., 2017). The low expression of the ubiquitin E3 ligase Trc8 in resistant cancer cells (MDR) that overexpress P-glycoprotein (Pgp) and multidrug resistance-related protein 1 (MRP1) leads to dysregulated cholesterol metabolism, decreased ubiquitination of HMGCR, increased cholesterol synthesis and storage, and the progression of colorectal cancer cells (Gelsomino et al., 2013).

Squalene epoxidase (SQLE) is a rate-limiting enzyme involved in cholesterol synthesis. Overexpression of SQLE impairs angiogenesis; however, angiogenesis is also attenuated when SQLE is silenced. The ERAD-associated ubiquitin ligase MARCH6 regulates endothelial cholesterol homeostasis by promoting SQLE degradation (Tan et al., 2020). Moreover, the E3 ligase MYLIP regulates cholesterol uptake by ubiquitinating LDL receptors, promoting LDLR degradation and blocking cholesterol uptake (Zelcer et al., 2009).

The effects of ubiquitination and deubiquitination on tumor metabolism are complex and require further study. The E3 ubiquitin ligase/DUB substrate network regulates tumor processes and is influenced by the cell type and environment for executive function. Understanding ubiquitination and SUMOylation in cancer metabolism is crucial to identifying new targets for cancer therapy. For example, the use of novel ubiquitin proteasome inhibitors to treat multiple myeloma is a therapeutic approach that links the ubiquitin system to the treatment of tumorigenesis and metabolic diseases. With the continuous development of proteomics technology, researchers have been able to accurately target and track ubiquitin-dependent tumorigenesis and diseases and develop more therapeutic targets.

## Ubiquitination and SUMOylation in Amino Acid Metabolism in Cancer

**FIGURE 5:**
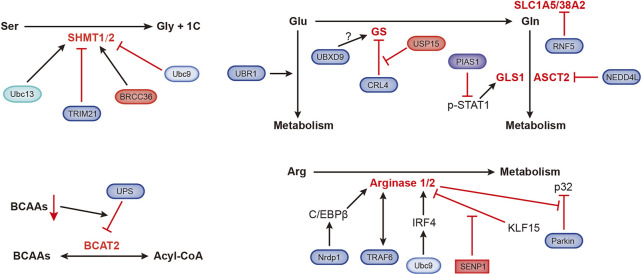
Ubiquitination- and SUMOylation-mediated regulation of amino acid metabolism. The black and red arrows represent activation and inhibition, respectively. ASCT2, alanine, serine, cysteine-preferring transporter 2; BCAAs, branched-chain amino acids; BCAT2, branched-chain amino acid transaminase 2; BRCC36, Zn^2+^-dependent deubiquitinating enzyme; CRL4, Cullin-RING ubiquitin ligase 4; GLS1, glutaminase 1; GS, glutamine synthetase; IRF4, interferon regulatory factor 4; KLF15, Kruppel-like factor 15; NEDD4L, NEDD4-like E3 ubiquitin protein ligase; Nrdp1, ring finger protein 41; PIAS1, protein inhibitor of activated STAT1; RNF5, ring finger protein 5; SHMT1/2, serine hydroxymethyltransferase 1/2; SLC1A5/38A2, L-glutamine carrier proteins; Ubc9, ubiquitin-conjugating enzyme; Ubc13, SUMO E2-conjugating enzyme; UBR1, ubiquitin protein ligase E3 component n-recognin 1; UBXD9, UBX domain containing 9; USP15, ubiquitin-specific protease 15.

Amino acids are materials required for protein synthesis in organisms. Tumor cells grow rapidly and require a large amino acid supply, which leads to the reprogramming of amino acid metabolism in tumor cells (Figure 5).

Organic groups containing only one carbon atom, called one-carbon units, produced during amino acid catabolism, are involved in important nucleotide synthesis and methylation in cells. Serine hydroxymethyltransferase (SHMT) catalyzes the metabolism of serine to produce one-carbon units and glycine, supporting rapidly proliferation of tumor cells. SHMT1 can be ubiquitinated and SUMOylated at the same lysine residue, Ubc13-mediated ubiquitination is required for SHMT1 nuclear export and stabilization, and Ubc9-mediated SUMOylation promotes SHMT1 nuclear degradation (Anderson et al., 2012). Serine hydroxymethyltransferase 2 (SHMT2) catalyzes the conversion of serine and glycine to support the proliferation of cancer cells. TRIM21 promotes the ubiquitination and degradation of SHMT2 in a glucose-dependent manner (Wei et al., 2018). BRCC36 is a deubiquitinating subunit that forms the BRISC complex. ABRO1, another subunit of the BRISC complex, binds to SHMT2α to prevent deubiquitination of the latter. The BRISC-bound SHMT2α is catalytically inactive, which inhibits SHMT2α function (Rabl et al., 2019).

The E3 ubiquitin ligase UBR1 regulates glutamate metabolism, which is essential for neuronal development and signal transduction (Chitturi et al., 2018). Glutamine (Gln) dependence is a characteristic of tumor metabolism, and the ubiquitin ligase RNF5 regulates the expression of the glutamine carrier proteins SLC1A5 and SLC38A2. Paclitaxel promotes the ubiquitination and subsequent degradation of SLC1A5/38A2 by RNF5 (Jeon et al., 2015). The E3 ligase NEDD4L inhibits mitochondrial metabolism by reducing the level of the glutamine transporter ASCT2 (Lee et al., 2020). Transcription of glutaminase (GLS1) is coactivated by p-STAT1 and p300. PIAS1 is an E3 SUMO ligase that inhibits activated STAT1 (p-STAT1). Kr-POK interacts with PIAS1, disrupting the interaction between PIAS1 and p-STAT1 and subsequently increasing the activity of p-STAT1 to promote GLS1 transcription. This process promotes cell growth, migration and motility (Hur et al., 2017; Li et al., 2020). Glutamine synthetase (GS) is the endogenous substrate of Cullin-RING ubiquitin ligase 4 (CRL4). When the extracellular glutamine concentration is high, acetylated GS is ubiquitinated by CRL4 and then degraded by the proteasome (Nguyen et al., 2016). Valosin-containing protein (VCP)/p97 promotes the degradation of ubiquitylated GS (Nguyen et al., 2017). p97 also binds to UBXD9, a member of the UBXD protein family. GS type III has been identified as a new interacting partner of UBXD9, but the specific regulatory mechanism is still unknown (Riehl et al., 2021). A recent study showed that USP15 antagonizes CRL4-mediated ubiquitination of GS and prevents GS degradation. USP15 is expressed at high levels in immunomodulatory drug (IMiD)-resistant cells, and the loss of USP15 renders these resistant cells sensitive to IMiD (Nguyen, 2021).

Arginase is very important in arginine metabolism, catalyzing the conversion of arginine into urea. Nrdp1 mediates the ubiquitination of the transcription factor C/EBPβ at the K63 site, which promotes the activation of C/EBPβ and subsequently upregulates arginase 1 (Arg1) expression (Ye et al., 2012). TRAF6 and arginase 1 are related, and they are expressed at high levels in myeloid-derived suppressor cells (MDSCs) (Song et al., 2021). Ubc9-mediated SUMOylation of interferon regulatory factor 4 (IRF4) increases its nuclear localization and stability. IRF4 induces the transcription of Arg1 and promotes the progression of the macrophage M2 program (Wang F. et al., 2019). KLF15 (Kruppel-like factor 15) binds to the Arg2 (arginase 2) promoter, which hinders Arg2 transcription. Under hypoxic conditions, SENP1-mediated deSUMOylation of KLF15 leads to the translocation of KLF15 from the nucleus to the cytoplasm, which induces the expression of Arg2 (Pandey et al., 2018). Arg2 was recently shown to regulate Parkin-dependent p32 degradation, promoting Ca^2+^-dependent eNOS activation (Koo et al., 2021).

The level of branched-chain amino acids (BCAAs) in plasma is related to the risk of pancreatic cancer. Branched-chain amino acid transaminase 2 (BCAT2) reversibly catalyzes BCAA degradation to branched-chain acyl-CoA. BCAA deprivation stimulates the degradation of acetylated BCAT2 through by the ubiquitin–proteasomeubiquitin-proteasome pathway (Lei et al., 2020).

## Conclusion

Similar to the human resources (HR) department in a company, which hires, trains, assigns work or fires personnel to make this huge machine (company) run normally, the cell is a more complex machine, and its signal transduction is more ingenious and delicate. Ubiquitination and SUMOylation are the HR of cells. They determine the fates of proteins and cellular signal transduction and determine the fate of the entire cell. Metabolic reprogramming occurs in various diseases, and an increasing number of studies are explaining the relationship between metabolism and diseases, especially cancer.

The regulation of ubiquitination and SUMOylation are similar in many respects. They both modify the substrate protein, and the modification binds covalently to the lysine residues of these proteins to determine the fate of the proteins. The motifs they recognize are different. Few proteins are involved in regulating ubiquitination and SUMOylation, but these processes both regulate a large number of proteins. Ubiquitin and SUMO proteins only show 18% sequence similarity, but their structures are similar. The main difference is that SUMO proteins have a long and flexible N-terminal extension. Compared with ubiquitination, SUMOylation is a relatively simple process. As described in the present review, SUMOylation also regulates fewer metabolic processes.

Here, we summarize the research on how ubiquitination and SUMOylation affect metabolic reprogramming. Based on the results from these recent studies, we realize that ubiquitination, SUMOylation and metabolic reprogramming are closely related. Neither is an independent process, and their effects are mutual. Ubiquitination and SUMOylation reshape metabolism by affecting important regulatory factors in the metabolic process (Lavie et al., 2018). The altered metabolic substrate level in metabolic reprogramming also affects the processes of ubiquitination and SUMOylation (Selak et al., 2005). Although many studies have been conducted in this field, research on SUMOylation in metabolic reprogramming is lacking. As stated in a recent review, SUMO: from bench to bedside (Chang and Yeh, 2020), research on SUMOylation in metabolic reprogramming must be a focus of future studies. We noticed that epigenetic regulation is also involved, increasing the complexity (Wang T. et al., 2019). Ubiquitination and SUMOylation are also involved in other cellular signal transduction pathways, including autophagy and ferroptosis (Varshavsky, 2017; Chen X. et al., 2021). These regulatory mechanisms are still unknown. Research designed to understand the complex regulatory mechanism must continue.

Ubiquitination and SUMOylation may be the direction of targeted therapy. An understanding of their relationship with cancer metabolism supports the development of new treatment strategies. Future research should also focus on treatment strategies that correct abnormal ubiquitination and SUMOylation. The development of new chemotherapeutic drugs targeting ubiquitination and/or SUMOylation combined with immunotherapy may overcome the limitations of chemotherapy and immunotherapy. This approach shows great promise and will provide benefits to human health.
